# Synthetic bakuchiol derivatives: ester and ether analogs with activity against clinically important bacteria

**DOI:** 10.3389/fphar.2025.1619997

**Published:** 2025-10-27

**Authors:** Francisca Valdés, Evelyn Muñoz, Manuel Martinez, Catalina Ferreira, Valentina Silva, Alejandro Madrid, Katy Díaz, Constanza Villarroel, Iván Montenegro

**Affiliations:** ^1^ Laboratory of Natural Products and Organic Synthesis (LPNSO), Department of Science and Geography, Faculty of Natural and Exact Sciences, Universidad de Playa Ancha, Valparaíso, Chile; ^2^ Department of Chemistry, Universidad Técnica Federico Santa María, Valparaíso, Chile; ^3^ Millennium Nucleus Bioproducts, Genomics and Environmental Microbiology (BioGEM), Valparaíso, Chile; ^4^ Center of Interdisciplinary Biomedical and Engineering Research for Health (MEDING), Escuela de Obstetricia y Puericultura, Facultad de Medicina, Universidad de Valparaíso, Valparaíso, Chile

**Keywords:** *Otholobium glandulosum*, bakuchiol, bakuchiol derivatives, antibacterial activity, clinical bacteria

## Abstract

**Introduction:**

With the rise of antibiotic resistance and healthcare-associated infections, there is a growing need for alternative therapies. *Otholobium glandulosum* (L.) J.W. Grimes (= *Psoralea glandulosa* L.) (Fabaceae) and its active compound, bakuchiol, have demonstrated significant antimicrobial and biological potential. This study investigates bakuchiol-based synthetic derivatives as promising antibacterial agents against clinically relevant pathogens.

**Methods:**

From the aerial parts of *O. glandulosum*, a resinous exudate was obtained, from which bakuchiol was isolated. This compound was used as a precursor to synthesize a series of ester-type (4–8) and ether-type (9–15) derivatives. All compounds were purified, their structures were confirmed by nuclear magnetic resonance (NMR), and they were evaluated *in vitro* for antibacterial activity against Gram-positive and Gram-negative strains. The most active derivatives were further tested in live/dead assays, and their pharmacokinetic and toxicity profiles were predicted *in silico* using the SwissADME and ADMETlab servers.

**Results:**

The ester derivatives exhibited bactericidal activity against *Staphylococcus aureus* and *Streptococcus agalactiae*, with compounds 4 and 5 being particularly effective, causing 90% growth inhibition. Compound 6 displayed a minimum inhibitory concentration (MIC) of 320 μg/mL against *Pseudomonas aeruginosa*. However, none of the compounds showed bactericidal activity against *Escherichia coli*. A structure–activity relationship (SAR) analysis indicated that chain length, lipophilicity, and stereochemistry influenced both efficacy and bacterial selectivity. *In silico* assays indicated acceptable absorption, low mutagenicity, and moderate hepatotoxicity, with limitations related to high LogP values.

**Conclusion:**

These results support the potential of bakuchiol ester-type derivatives as antibacterial agents, which encourages future *in vivo* studies and synthetic optimization.

## 1 Introduction

Over the past four decades, the global etiology of healthcare-associated infections (HCAIs) has evolved considerably. A clear example is sepsis, which is mainly caused by bacteria transmitted within health facilities. These infections constitute a major health problem, contributing to increased morbidity and mortality, prolonged hospital stays, and increased direct and indirect healthcare costs (Vázquez-Cruz et al., 2018). Given the widespread problem of antibiotic resistance and the potential side effects associated with pharmacological treatments, it is imperative to explore innovative therapeutic alternatives that are less likely to cause complications (Chaachouay and Lahcen, 2024). One of these alternatives is the use of plant extracts or resinous exudates, which constitute a vast chemical arsenal, of which only approximately one-third has been characterized (Khushboo et al., 2010). In parallel, synthetic derivatives obtained through specific chemical synthesis reactions have been developed with the purpose of generating bioactive compounds with desirable biological properties (Li et al., 2021).

**FIGURE 1:**
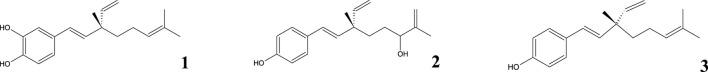
Natural compounds 1–3 isolated from *Otholobium glandulosum*.

*Otholobium glandulosum* (L.) J.W. Grimes, also known as *Psoralea glandulosa* L. or popularly as “culén,” belongs to the Fabaceae family. This shrub is characterized by producing resinous exudates from the glandular trichomes that cover the surface of its leaves and stems (Madrid and Silva, 2024). It has traditionally been used as a cicatrizant, anti-hemorrhoidal, and antiseptic for treating bacterial and fungal infections and skin diseases (Krishna et al., 2022). Its resinous leaves are rich in multiple bioactive compounds, such as 3-hydroxy-bakuchiol (1), 12-hydroxy-bakuchiol (2), and bakuchiol (3) (see Figure 1) (Backhouse et al., 2001; Madrid et al., 2012a; Madrid et al., 2012b; Madrid et al., 2015a).

Since its discovery and isolation in 1966, bakuchiol and its chemically modified analogs have been reported to exhibit a wide range of biological activities (Labbé et al., 1996). Among these, its potential anticancer effect has been highlighted, as evidenced by its ability to inhibit growth and induce apoptosis in melanoma cells (Madrid et al., 2015a). In addition, bakuchiol has been shown to prevent mitochondrial lipid peroxidation and to protect enzymes from oxidative stress (Krishna et al., 2022). Its antiviral activity has also been reported (Shoji et al., 2015). Moreover, it has significant antimicrobial effects, with reports of inhibiting the growth of *Streptococcus mutans* and *Actinomyces viscosus*, which reflects strong antibacterial potential (Koul et al., 2019). These findings position bakuchiol as a promising agent for controlling bacterial diseases (Madrid and Silva, 2024).

Considering this background, we designed two series of syntheses using bakuchiol as a base molecule, substituting the phenolic hydroxyl group to obtain synthetic derivatives. This approach represents an innovative strategy to address the prevention and control of infections from clinically important bacteria worldwide, such as *Streptococcus agalactiae*, *Staphylococcus aureus*, *Pseudomonas aeruginosa*, and *Escherichia coli*. The antibacterial potential of bakuchiol (3) and its synthetic derivatives (4–15) was evaluated *in vitro* to determine their ability to disrupt membrane permeability and induce bacterial cell death.

## 2 Materials and methods

### 2.1 General

All chemicals and positive controls were obtained from Sigma-Aldrich (St. Louis, MO, United States). All reactions were monitored by thin-layer chromatography (TLC) on TLC pre-coated silica gel 60 F^25^4 (Merck KGaA, Darmstadt, Germany). Flash column chromatography (CC) was performed on silica gel (200–300 mesh) (Merck KGaA, Darmstadt, Germany). The ^1^H and ^13^C spectra were recorded in CDCl_3_ solutions and are referenced to the residual peaks of CHCl_3_ at *δ* = 7.26 ppm and *δ* = 77.0 ppm for ^1^H and ^13^C, respectively, on an Avance 400 Digital NMR spectrometer (Bruker, Rheinstetten, Germany) operating at 400.1 MHz for ^1^H and 100.6 MHz for ^13^C.

### 2.2 *Otholobium glandulosum*: Extraction of the resinous exudate and isolation of bakuchiol

Aerial parts of *O. glandulosum* were collected from “Las Dichas” (Route F-840; 33°17′27″S, 71°30′24″W), Casablanca, Valparaíso Region, Chile. The taxonomic identification was made by the botanist Patricio Novoa. The voucher specimen (N° Pg-11123) was deposited at the VALP Herbarium, Department of Biology, Universidad de Playa Ancha, Valparaíso, Chile.

The resinous exudate was obtained from *O. glandulosum* as previously described by Madrid et al. (2015b). Briefly, the method consisted of immersing fresh branches and leaves of *O. glandulosum* (2 kg) in dichloromethane for 30 s, followed by solvent removal under reduced pressure to obtain the resinous exudate. Ten grams of the resinous exudate were fractionated by silica gel CC using hexane–ethyl acetate with increasing polarity. Compound 3 was isolated with a yield of 3.61%, and its purity (95%) was confirmed by analytical HPLC. This procedure yielded the necessary mass for the development of synthetic derivatives 4–15. Isolated compound 3 was identified by comparing its spectroscopic data with previously described compounds in the literature (Labbé et al., 1996; Madrid et al., 2015b).

### 2.3 Synthesis and yields of bakuchiol synthetic derivatives

#### 2.3.1 General protocol for the synthesis of bakuchiol esters 4–8

Bakuchiol esters were synthesized in a 100-mL round-bottom flask; bakuchiol (1.0 mmol) was mixed with each respective anhydride (1.0 mmol), DMAP (0.01 mmol), and one drop of pyridine in dichloromethane (10 mL). The reaction mixture was stirred at room temperature for 30 min. The progress of the reaction was monitored by TLC until completion. After completion, the reaction mixture was neutralized with saturated aqueous KHSO_4_, washed with water, and extracted with ethyl acetate (3 × 25 mL). The resulting organic phase was dried over anhydrous sodium sulfate and filtered. The solvent was then evaporated under reduced pressure.

All compounds were separated and purified by CC eluting with mixtures of hexane/ethyl acetate of increasing polarity (9.0:1.0 →5.8:4.2). The progress in the separation of the derivatives was analyzed by TLC. The structural determination of synthetic derivatives was confirmed from their spectroscopic properties by NMR, and the purity of the compounds (96%) was confirmed by analytical HPLC. Compounds 4, 5, 7, and 8 were contrasted with findings previously reported in the literature (Majeed et al., 2012; Madrid et al., 2015b), and the details are available in the Supplementary Material. The new compound (6) is described below.

4-[(1*E*,3*S*)-3,7-dimethyl-3-vinylocta-1,6-dien-1-yl]phenyl pivalate (6): the compound was isolated as a white solid in a yield of 12.5%. ^1^H NMR (400 MHz, CDCl_3_): *δ* 7.35 (m, 2H, H-3 and H-5); 6.97 (m, 2H, H-2 and H-6); 6.30 (d, *J* = 16.3 Hz, 1H, H-7); 6.15 (d, *J* = 16.3 Hz, 1H, H-8); 5.88 (dd, *J* = 10.7 and 17.2 Hz, 1H, H-17); 5.11 (t, *J* = 7.0, 1H, H-12); 5.03 (m, 2H, H-18); 1.95 (m, 2H, H-11); 1.68 (s, 3H, H-14); 1.58 (s, 3H, H-15); 1.50 (m, 2H, H-10); 1.35 (s, 9H (CH_3_)_3_CO); 1.22 (s*,* 3H, H-16). ^13^C NMR (100 MHz, CDCl_3_): *δ* 177.1 ((CH_3_)_3_CO); 150.0 (C-5); 145.7 (C-17); 138.0 (C-8); 135.4 (C-1); 131.4 (C-13); 126.9 (C-2 and C-6); 126.3 (C-7); 124.7 (C-12); 121.5 (C-3 and C-5); 112.1 (C-18); 42.6 (C-9); 41.2 (C-10); 39.0 (C-2′); 27.1 (C-3′, C-4′ and C-5′); 25.7 (C-16); 23.2 (C-11 and C-15); 17.6 (C-14).

#### 2.3.2 General protocol for the synthesis of bakuchiol esters 9–15

Bakuchiol (1.1 mmol) was reacted with a series of alkyl halides (1.2 mmol each) in the presence of K_2_CO_3_ (1.5 mmol) in acetone (10 mL) and refluxed at 75 °C for 6 h. The reaction’s completion was verified by TLC, and the mixture was then poured into ice water (20 mL) and extracted with ethyl acetate (3 × 25 mL). The resulting organic phase was dried over anhydrous sodium sulfate and filtered. The solvent was then evaporated under reduced pressure.

All compounds were separated and purified by CC, eluting with mixtures of hexane/ethyl acetate of increasing polarity (9:1, 8:2, 7:3, and 6:4). The separated derivatives’ purity was confirmed via TLC analysis. NMR spectroscopy was used to confirm the molecular structure of the synthesized derivatives, and the purity of the compounds (96%) was confirmed by analytical HPLC. The spectroscopic data for compound 9 were found to be in agreement with previously reported values (Majeed et al., 2012; Madrid et al., 2015a). The new compounds 10–15 are described below.

Allyl 4-[(1E, 3S)-3,7-dimethyl-3-vinylocta-1,6-dien-1-yl]phenyl ether (10): the compound was isolated as a white solid in a yield of 34.4%. ^1^H NMR (400 MHz, CDCl_3_): *δ* 7.29 (d, *J* = 8.7 Hz, 2H, H-2, and H-6); 6.85 (d, *J* = 8.7 Hz, 2H, H-3, and H-5); 6.26 (d, *J* = 16.2 Hz, 1H, and H-7); 6.05 (m, 2H, H-8, and H-2′); 5.88 (dd, *J* = 10.9 and 16.3 Hz, 1H, and H-17); 5.43 (d, *J* = 1.4 Hz, 1H, and H-3b′); 5.38 (d, *J* = 1.4 Hz, 1H, and H-3a′); 5.11 (t, *J* = 7.0 Hz, 1H, and H-12); 5.02 (m, 2H, and H-18); 4.53 (d, *J* = 5.3 Hz, 2H, and H-1′); 1.95 (m, 2H, and H-11); 1.67 (s, 3H, and H-14); 1.57 (s, 3H, and H-15), 1.47 (m, 2H, and H-10), 1.19 (s, 3H, and H-16). ^13^C NMR (100 MHz, CDCl_3_): *δ* 157.7 (C-4); 146.0 (C-17); 135.9 (C-7); 133.3 (C-8); 131.3 (C-2′); 130.8 (C-13); 127.1 (C-2 and C-6); 126.5 (C-1); 124.8 (C-12); 117.6 (C-3′); 114.7 (C-3 and C-5); 111.9 (C-18); 68.8 (C-1′); 42.5 (C-9); 41.3 (C-10); 25.7 (C-16); 23.3 (C-15); 23.2 (C-11); 17.6 (C-14).

4-[(1E, 3S)-3,7-dimethyl-3-vinylocta-1,6-dien-1-yl]phenyl 2-methylprop-2-en-1-yl ether (11): the compound was isolated as a white solid in a yield of 68.9%. ^1^H NMR (400 MHz, CDCl_3_): *δ* 7.28 (d, *J* = 8.7 Hz, 2H, H-2, and H-6); 6.85 (d, *J* = 8.7 Hz, 2H, H-3, and H-5); 6.26 (d, *J* = 16.2 Hz, 1H, and H-7); 6.06 (d, *J* = 16.2 Hz, 1H, and H-8); 5.88 (dd, *J* = 10.9 and 16.3 Hz, 1H, and H-17); 5.10 (t, *J* = 7.0, 1H, and H-12), 5.03 (m, 2H, and H-18), 4.98 (s, 2H, and H-3′); 4.43 (s, 2H, and H-1′); 1.95 (m, 2H, and H-11); 1.82 (s, 3H, and H-4′); 1.67 (s, 3H, and H-14); 1.57 (s, 3H, and H-15), 1.47 (m, 2H, and H-10), 1.19 (s, 3H, and H-16). ^13^C NMR (100 MHz, CDCl_3_): *δ* 157.9 (C-4); 145.6 (C-17); 140.9 (C-2′); 135.8 (C-7); 131.3 (C-8); 130.8 (C-13); 127.1 (C-2 and C-6); 126.5 (C-1); 124.80 (C-12); 114.8 (C-3 and C-5); 112.7 (C-4′); 111.8 (C-18); 71.7 (C-1′); 42.5 (C-9); 41.3 (C-10); 25.7 (C-16); 23.3 (C-15); 23.2 (C-11); 19.4 (C-3′); 17.6 (C-14).

(2*E*)-but-2-en-1-yl 4-[(1E, 3S)-3,7-dimethyl-3-vinylocta-1,6-dien-1-yl]phenyl ether (12): the compound was isolated as a white solid in a yield of 29.8%. ^1^H NMR (400 MHz, CDCl_3_): *δ* 7.29 (d, *J* = 8.7 Hz, 2H, H-2, and H-6); 6.85 (d, *J* = 8.7 Hz, 2H, H-3, and H-5); 6.26 (d, *J* = 16.2 Hz, 1H, and H-7); 6.06 (d, *J* = 16.2 Hz, 1H, and H-8); 5.87 (dd, *J* = 10.9 and 16.3 Hz, 1H, and H-17); 5.86 (m, 1H, and H-2′); 5.73 (m, 1H, and H-3′); 5.11 (t, *J* = 7.0, 1H, and H-12), 5.03 (m, 2H, and H-18); 4.45 (d, *J* = 6.0 Hz, 2H, and H-1′); 1.95 (m, 2H, and H-11); 1.75 (s, 3H, and H-4′); 1.67 (s, 3H, and H-14); 1.58 (s, 3H, and H-15); 1.49 (m, 2H, and H-10); 1.26 (s, 3H, and H-16). ^13^C NMR (100 MHz, CDCl_3_): *δ* 157.8 (C-4); 146.0 (C-17); 135.7 (C-7); 131.3 (C-8); 130.7 (C-13); 130.6 (C-2′); 127.1 (C-2 and C-6); 126.5 (C-1); 126.0 (C-3′); 124.8 (C-12); 114.7 (C-3 and C-5); 111.8 (C-18); 68.7 (C-1′); 42.5 (C-9); 41.3 (C-10); 25.7 (C-16); 23.3 (C-15); 23.2 (C-11); 19.4 (C-3′); 17.84 (C-14); 17.6 (C-4′).

4-[(1*E*,3*S*)-3,7-dimethyl-3-vinylocta-1,6-dien-1-yl]phenyl (2*E*,6*E*)-3,7,11-trimethyldodeca-2,6,10-trien-1-yl ether (13): the compound was isolated as a white solid in a yield of 15.7%. ^1^H NMR (400 MHz, CDCl_3_): *δ* 7.28 (d, *J* = 8.7 Hz, 2H, H-2, and H-6); 6.85 (d, *J* = 8.7 Hz, 2H, H-3, and H-5); 6.26 (d, *J* = 16.2 Hz, 1H, and H-7); 6.06 (d, *J* = 16.2 Hz, 1H, and H-8); 5.87 (dd, *J* = 10.9 and 16.3 Hz, 1H, and H-17); 5.49 (m, 1H, and H-2′); 5.10 (m, 3H, H-12, H-6′, and H-10′); 5.02 (m, 2H, and H-18); 4.53 (d, *J* = 6.5 Hz, 2H, and H-1′); 2.10 (m*,* 6H, H-5′, H-8′, and H-9′); 1.96 (m, 4H, H-11, and H-4′); 1.73 (s, 3H, and H-13′); 1.71 (s, 6H, H-14′, and H-15′); 1.68 (s, 6H, H-14, and H-12′); 1.59 (s, 3H, and H-15); 1.49 (m, 2H, and H-10); 1.20 (s, 3H, and H-16). ^13^C NMR (100 MHz, CDCl_3_):*δ* 158.3 (C-4); 146.0 (C-17); 141.1 (C-3′); 135.7 (C-7); 135.4 (C-8); 131.3 (C-7′ and C-11′); 130.6 (C-13); 127.1 (C-2 and C-6); 126.6 (C-1); 124.8 (C-12); 124.3 (C-6′); 123.7 (C-10′); 119.5 (C-2′); 114.7 (C-3 and C-5); 111.8 (C-18); 64.9 (C-1′); 42.5 (C-19); 41.3 (C-10); 39.7 (C-4′ and C-8′); 26.7 (C-9′); 26.2 (C-5′); 25.7 (C-16 and C-12′); 23.3 (C-15); 23.2 (C-11); 17.7 (C-14); 17.62 (C-15′); 16.7 (C-13′); 16.0 (C-14′).

4-[(1E, 3S)-3,7-dimethyl-3-vinylocta-1,6-dien-1-yl]phenyl 3-methylbut-2-en-1-yl ether (14): the compound was isolated as a white solid in a yield of 26.8%. ^1^H NMR (400 MHz, CDCl_3_): *δ* 7.29 (d, *J* = 8.7 Hz, 2H, H-2, and H-6); 6.85 (d, *J* = 8.7 Hz, 2H, H-3, and H-5); 6.26 (d, *J* = 16.2 Hz, 1H, and H-7); 6.06 (d, *J* = 16.2 Hz, 1H, and H-8); 5.87 (dd, *J* = 10.9 and 16.3 Hz, 1H, and H-17); 5.49 (t, *J* = 10.8 Hz, 1H, and H-2′); 5.11 (t, *J* = 7.1 Hz, 1H, and H-12); 5.02 (m, 2H, and H-18); 4.50 (d, *J* = 6.7 Hz, 2H, and H-1′); 1.95 (m, 2H, and H-11); 1.79 (s, 3H, and H-4′); 1.74 (s, 3H, and H-5′); 1.67 (s, 3H, and H-14); 1.58 (s, 3H, and H-15); 1.49 (m, 2H, and H-10); 1.19 (s, 3H, and H-16). ^13^C NMR (100 MHz, CDCl_3_): *δ* 158.0 (C-4); 146.0 (C-17); 138.1 (C-3′); 135.7 (C-7); 131.3 (C-8); 130.6 (C-13); 127.1 (C-2 and C-6); 126.6 (C-1); 124.8 (C-12); 119.7 (C-2′); 114.7 (C-3 and C-5); 111.8 (C-18); 64.8 (C-1′); 42.5 (C-19); 41.3 (C-10); 25.8 (C-4′); 25.7 (C-14); 23.4 (C-15); 23.2 (C-11); 18.2 (C-5′); 17.6 (C-16).

(2*E*)-3,7-dimethylocta-2,6-dien-1-yl 4-[(1*E*,3*S*)-3,7-dimethyl-3-vinylocta-1,6-dien-1-yl]phenyl ether (15): the compound was isolated as a white solid in a yield of 21.5%. ^1^H NMR (400 MHz, CDCl_3_): *δ* 7.27 (d, *J* = 8.7 Hz, 2H, H-2, and H-6); 6.85 (d, *J* = 8.7 Hz, 2H, H-3, and H-5); 6.26 (d, *J* = 16.2 Hz, 1H, and H-7); 6.06 (d, *J* = 16.2 Hz, 1H, and H-8); 5.87 (dd, *J* = 10.9 and 16.3 Hz, 1H, and H-17); 5.49 (m, 1H, and H-2′); 5.10 (m, 2H, H-12, and H-6′); 5.02 (m, 2H, and H-18); 4.53 (d, *J* = 6.5 Hz, 2H, and H-1′); 2.10 (m*,* 4H, H-4′, and H-5′); 1.95 (m, 2H, and H-11); 1.73 (s, 3H, and H-9′); 1.68 (s, 6H, H-14, and H-8′); 1.59 (s, 3H, and H-10′); 1.49 (m, 2H, and H-10); 1.19 (s, 3H, and H-16). ^13^C NMR (100 MHz, CDCl_3_): *δ* 158.0 (C-4); 146.0 (C-17); 141.1 (C-3′); 135.7 (C-7); 131.8 (C-8); 131.3 (C-7′); 130.6 (C-13); 127.1 (C-2 and C-6); 126.6 (C-1); 124.8 (C-12); 123.8 (C-6′); 119.5 (C-2′); 114.7 (C-3 and C-5); 111.8 (C-18); 64.9 (C-1′); 42.5 (C-19); 41.3 (C-10); 39.5 (C-4′); 29.7 (C-16); 26.3 (C-5′); 25.7 (C-15); 23.4 (C-8′); 23.2 (C-11); 17.7 (C-10′); 17.6 (C-14); 16.6 (C-9′).

### 2.4 Antibacterial activity determination

The bacteria used for the biological tests were obtained from the Clinical Microbiology Laboratory of Dr. Patricio Godoy Martinez at the Universidad Austral de Chile (UACH). The strains used were the Gram-positive *S. aureus* ATCC 25923 and *S. agalactiae* ATCC 27956, and the Gram-negative *E. coli* ATCC 25922 and *P. aeruginosa* from the Bank of the Institute of Clinical Microbiology, UACH. The evaluations were carried out at the Biological Testing Laboratory, Department of Chemistry, Universidad Técnica Federico Santa María, under the supervision of Dr. Katy Díaz Peralta.

The minimum inhibitory concentration (MIC) of the synthetic derivatives was determined using the protocol mentioned in the literature (Díaz et al., 2018), with minor modifications to the broth dilution method for each of the series evaluated. Ciprofloxacin™ and meropenem™ were used as positive controls, and the same concentrations and evaluation conditions were applied as described earlier. To obtain the effective concentration (EC_50_) of each compound, the percentage of inhibition for each treatment and concentration was fit to a dose–response equation (Olea et al., 2019). The analysis of the fit was performed with Origin 8.0 software. Microsoft Excel 365™ software (Microsoft Corporation, Redmond, WA, United States) was used for statistical analysis, applying a one-way ANOVA and Tukey’s test to observe the relationship between the synthesized compounds. A *p*-value <0.05 was used to evaluate the statistical significance of the data.

The protocol for the live/dead viability assay, as previously described by Berney et al. (2007), was adapted for this study with necessary adjustments to accommodate the specific compounds being tested. The assay was performed on the following bacterial strains: *P. aeruginosa*, *S. aureus*, and *S. agalactiae*. The commercially available LIVE/DEAD BacLight Kit (Invitrogen), which contains the stains propidium iodide (PI) and SYTO9, was used. A 1:1 mixture of the two dyes was prepared (Boulos et al., 1999). Subsequently, 3 µL of this mixture was added to each Eppendorf tube containing the bacterial sample, ensuring a final DMSO concentration of 0.3%. The samples were then incubated for 15 min in the dark. For observation, a 10-µL aliquot of the sample was mounted on a slide, covered with a coverslip, and sealed with 10 µL of BacLight™ mounting oil to preserve the preparation. Visualization was performed using a Leica DM500 fluorescence microscope equipped with a Leica ICC50 HD camera. Images were captured using Leica I3 and N2.1 cube filters, with emissions at 450 nm–490 nm and 515 nm–560 nm, respectively. The compounds with the highest biological activity against the evaluated pathogens were selected for this test. The concentration used was determined by the MIC of each compound.

### 2.5 *In silico* assays

For obtaining the pharmacokinetic and toxicological parameters, the chemical structures of the analyzed compounds in the SMILES format were used on the SwissADME platform (http://www.swissadme.ch/) and ADMETlab 2.0 (https://ai-druglab.smu.edu/).

## 3 Results and discussion

### 3.1 Synthesis of derivatives

**SCHEME 1:**
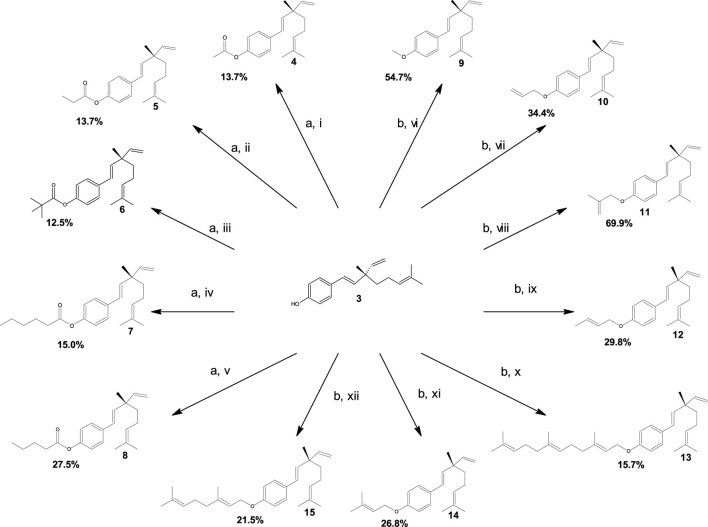
Synthesis and yields of compounds 4–15. Reagents and conditions: **(a)** 4-dimethylaminopyridine, pyridine, and dichloromethane; stirring at room temperature for 30 min; (i) acetic anhydride, (ii) propionic anhydride, (iii) trimethylacetic anhydride, (iv) hexanoic anhydride, and (v) valeric anhydride. **(b)** K_2_CO_3_ and acetone; reflux at 75 °C for 6 h; (vi) iodomethane, (vii) allyl bromide, (viii) 3-bromo-2-methylpropene, (ix) crotyl bromide, (x) farnesyl bromide, (xi) prenyl bromide, and (xii) geranyl bromide.

From natural meroterpenoid 3, five ester-type derivatives 4–8 and seven ether-type derivatives 9–15 were synthesized. The synthetic strategies applied for the synthesis of both known and new molecules are described in Scheme 1.

Bakuchiol (3) was purified in analogy to the procedures described in the study by Madrid et al. (2012a), with small variations from the resinous exudate of *O. glandulosum*. The ester-type compounds were obtained with moderate yields (12.5%–27.5%) through the acyl nucleophilic substitution of compound 3 with appropriate anhydride in dichloromethane, using DMAP and pyridine as catalysts. On the other hand, ether-type compounds 9–15 were synthesized with moderate-to-good yields (15.7%–69.9%) via nucleophilic substitution of compound 3 with the suitable alkyl halide in acetone.

The structures of compounds 4–15 were established based on NMR techniques. In all the synthesized compounds, chemical shifts corresponding to the starting material, compound 3, were observed. In the ^13^C NMR experiments, the esterification of compounds 4–8 was confirmed by the shielding of phenolic carbon, which showed a chemical shift in the range of δ 150.0 to 149.5 ppm. This contrasts with the chemical shift observed for the phenolic carbon of the starting material, which is located at δ 154.6 ppm. The ester carboxyl group was observed in all ester-type compounds, with a δ range of 177.1 to 169.5 ppm. For the ether-type derivatives 9–15, the ^1^H NMR spectrum revealed resonances at δ 4.43 ppm–4.53 ppm, and the ^13^C NMR revealed resonances at δ 68.4 ppm–71.7 ppm. These signals were attributed to the protons of the O–CH_2_ group on the alkoxy chain that attaches to the ring. This is a characteristic of alkyl chains on aromatic rings that results from the alkylation reaction.

### 3.2 Antibacterial activity

All synthesized compounds 4–15 showed bactericidal activity against *P. aeruginosa*. Among them, derivatives 6 and 7 were the most potent. Specifically, compound 6 achieved 90% growth inhibition, whereas compound 7 achieved 94% inhibition. Both presented an MIC of 320 μg/mL and very similar EC_50_ values of 31.6 μg/mL and 30.74 μg/mL, respectively (Tables 1 and 2). In stark contrast, it is important to note that none of these synthetic derivatives demonstrated bactericidal activity against *E. coli*.

**TABLE 1:** MIC values (μg/mL) of bakuchiol (3) and its derivatives 4–15 against the bacteria under study.

Compound	*E. coli*		*P. aeruginosa*		*S. aureus*		*S. agalactiae*	
	MIC	% Inhibition	MIC	% Inhibition	MIC	% Inhibition	MIC	% Inhibition
3	>1,280	100	>1,280	99.6	40	99.6	80	100
4	>1,280	100	320	98	80	70	160	66.30
5	>1,280	81	320	90	160	60	320	90
6	>1,280	100	320	92	>1,280	100	>1,280	100
7	>1,280	72	320	94	320	74	320	82
8	>1,280	58	640	90	160	46	320	98
9	>1,280	69	>1,280	99.4	>1,280	73	>1,280	55
10	>1,280	78	>1,280	97	>1,280	81	>1,280	74
11	>1,280	79	>1,280	99.7	>1,280	77	>1,280	78
12	>1,280	76	>1,280	100	>1,280	62	>1,280	66
13	>1,280	96	>1,280	89.9	>1,280	85	>1,280	74
14	>1,280	91	>1,280	96.4	>1,280	71	>1,280	66
15	>1,280	78	>1,280	83.3	>1,280	69	>1,280	49
Ciprofloxacin	0.5	100	<0.5	99	<1.25	69	1.25	100
Meropenem	<0.5	100	0.5	100	<0.5	100	<0.5	99
DMSO	I	I	I	I	I	I	I	I

I, inactive.

**TABLE 2:** EC_50_ values (μg/mL) of bakuchiol (3) and its derivatives 4–15 against the bacteria under study.

Compound	CLogP^a^	*E. coli*	*P. aeruginosa*	*S. aureus*	*S. agalactiae*
3	6.40 ± 0.38	141 ± 3.34^*^	120.8 ± 2.51	24.2 ± 0.34^*^	<5
4^b^	6.38 ± 0.37	72 ± 1.19^*^	32.02 ± 2.93^**^	6.8 ± 2.33^*^	56.1 ± 2.25^*^
5^c^	6.91 ± 0.37	82.3 ± 1.97^*^	31.66 ± 3.41^*^	77.4 ± 2.71^*^	100.7 ± 2.20^*^
6^c^	7.60 ± 0.38	137.1 ± 1.87^*^	33.27 ± 3.29^**^	>640	210.5 ± 2.19^*^
7^b^	8.50 ± 0.37	68.8 ± 1.66^*^	30.71 ± 3.32^**^	113.3 ± 2.57^*^	125.7 ± 1.91^*^
8^c^	7.97 ± 0.37	105.2 ± 1.62^*^	32.02 ± 2.93^**^	113.3 ± 2.57^*^	125.7 ± 1.91^*^
9	6.88 ± 0.38	124.93 ± 2.62^*^	102.65 ± 2.11	109.74 ± 4.5^*^	242.25 ± 3,84
10	7.69 ± 0.39	167.63 ± 3.40^*^	122.94 ± 2.58	132.35 ± 3.79^*^	263.09 ± 3.53
11	8.21 ± 0.38	145.09 ± 3.04^*^	121.92 ± 2.40	117.78 ± 2.88^*^	254.54 ± 3.84
12	8.24 ± 0.40	156.4 ± 3.03^*^	136.49 ± 2.70	155.67 ± 4.07^*^	421.87 ± 3.24
13	8.77 ± 0.40	147.61 ± 2.87^*^	134.62 ± 3.54	98.51 ± 2.67^*^	300.52 ± 2.65
14	11.65 ± 0.43	134.15 ± 3.05^*^	139.99 ± 2.60	165.35 ± 4.36^*^	573.2 ± 3.01
15	13.64 ± 0.44	134.44 ± 2.81^*^	118.46 ± 2.28	89.88 ± 3.72^*^	313.3 ± 3.32
Ciprofloxacin	0.65 ± 1.44	<2.5	<2.5	<2.5	<2.5

^a^ CLogP = lipophilicity calculated with ACD/ChemSketch. Data were evaluated by one-way analysis of variance (ANOVA), followed by Tukey’s *post hoc* test to determine significant differences among the synthesized compounds. A significance level of *p < 0.05 and **p < 0.01.

^b^ Belonging to the same statistical group.

^c^ Belonging to independent statistical groups unrelated to each other.

The differing results from the *in vitro* assays for *E. coli* compared to those of other Gram-negative bacteria, such as *P. aeruginosa*, can be attributed to the significant structural differences in their cell walls. The peptidoglycan layer of *E. coli* is approximately twice as thick (6 µm) as that of *P. aeruginosa* (3 µm). Additionally, the length of the disaccharide chains in *E. coli* peptidoglycan is longer (20–35 units) than that in *P. aeruginosa* (16 units) (Vollmer and Seligman, 2010). These characteristics suggest a more significant physical impediment to the entry of the synthetic compounds into *E. coli*, compared to that in the other strains. This barrier is even more pronounced in Gram-positive bacteria, which possess an even thicker peptidoglycan layer, thus explaining the high activity of the derivatives against *S. aureus* and *S. agalactiae*.

It has been demonstrated that the esterification of ferulic acid enhances its antibacterial efficacy against *E. coli*, *P. aeruginosa*, *S. aureus*, and *Bacillus subtilis* (Song et al., 2023). Similarly, chemically synthesized PEGylated dopamine esters (PDE) exhibit antibacterial activity against *B. subtilis*, *S. aureus*, *P. aeruginosa*, and *Proteus vulgaris* (Jarial et al., 2018). This activity is attributed to the alkyl chains, which confer biologically active properties such as increased lipophilicity. This characteristic allows them to disrupt the cell membrane of microorganisms, thus facilitating their antibacterial action (Ngaini et al., 2012). Another factor that supports the absence of a bactericidal effect in *E. coli* is that this bacterium has esterases, which, by their nature, have specific cut sites (Ferrer et al., 2004).

Ester derivatives 4–8 evaluated in this study showed antibacterial activity against Gram-positive bacteria, including the inhibition of *S. agalactiae* and *S. aureus* growth (Table 1). Compounds 4 and 5 stood out for their significant bactericidal activity. Both compounds have similar chain lengths, differing by only one carbon atom, which is reflected in their partition coefficients (LogP) (Table 2). These data are crucial, as the other compounds, 6–8, despite having higher LogP values, showed reduced activity against both Gram-positive bacteria (Table 2).

From the perspective of ether derivatives 9–15, as initially indicated in the context of this study, a molecular modification was made to the bakuchiol structure based on what was presented in the literature. As evidenced in the study, all synthesized compounds showed promising antibacterial activity against *E. coli*, where the presence of these particular chains effectively contributed to the antibacterial activity. However, the inhibitory activity decreased with increasing chain length. This latter phenomenon may explain the inactivity of compound 15. From a structural point of view, the side chains or lipopolysaccharide O antigens (LPS) present in the outer membrane of Gram-negative bacteria and the teichoic acids in Gram-positive bacteria are very polar and negatively charged, and this is why they prevent the passage of large molecules into the bacterial cytoplasm, especially uncharged and lipophilic molecules, as is the case for these compounds (Vollmer and Seligman, 2010).

In addition, the inactivity of compounds 9–15 against *E. coli* can be explained by two main factors. First, their physicochemical properties, particularly their high lipophilicity (LogP from 6.88 to 13.6), could impede their passage through the bacterial cell wall, preventing them from reaching their intracellular target (Ovalle et al., 2017). Second, at the molecular level, it is likely that *E. coli* etherases, which are highly specific for substrates such as anhydro-N-acetylmuramic acid (Mazariego-Espinosa et al., 2010), do not recognize the structure of these synthetic derivatives. Delving deeper into the molecular interaction, the lack of activity could be due to steric hindrance. The long carbon chains at the ends of the molecules might physically block the enzyme’s access to the ether or ester functional group, thereby preventing the formation of the ligand–receptor complex (Reddy et al., 2010). This hypothesis is strengthened by the observation that substituting a part of the structure with a benzene ring and a phenol group restores activity. This suggests that the enzyme can recognize this new group as a binding or cleavage site, overcoming the structural blockage. Therefore, the high specificity of enzymes for a precise spatial orientation (Song et al., 2023) is a determining factor.

On the other hand, results from the Live/Dead BacLight™ bioassay revealed that ester derivatives 4–8, including both short- and long-chain variants, possess significant antibacterial activity. This activity was associated with direct damage to the cell membrane, as evidenced by the entry of PI into the cells. This finding confirms that the mechanism of action for these compounds involves the loss of membrane integrity. Although this is the primary effect observed, the possibility of other co-existing cell death mechanisms cannot be ruled out. This approach has been validated in similar studies, such as the one by Reddy et al. (2010), who exclusively used PI as a marker for damage and measured the bacterial population via flow cytometry to confirm membrane permeabilization caused by their bakuchiol derivatives.

To delve deeper into the mechanism of action beyond general membrane damage, the potency of each compound was quantified. The results highlighted compound 4 as the most effective derivative. In the study, bakuchiol was the least active compound, leaving 74% of the cells viable. In contrast, compound 4 demonstrated the greatest bactericidal effectiveness, reducing the *S. aureus* population to just 13% live cells. However, its efficacy was lower against *P. aeruginosa*, where cell viability remained at 65%. It is postulated that the broad-spectrum activity of compound 4—characterized by its low polarity and a terminal methyl group—is due to its spatial conformation. This structure could expose the benzene ring, allowing it to anchor in the active site of an enzyme and, thus, facilitating its recognition by an esterase.

In contrast to the most potent short-chain derivative, long-chain compounds 7 and 8 also showed notable activity patterns, albeit with different selectivity. Compound 7 was particularly effective against *P. aeruginosa*, reducing the viable population to 29%, whereas compound 8 showed its greatest activity against *S. aureus*, with 50% of the cells remaining viable. This suggests that even the difference of a single carbon in the chain influences antibacterial activity, likely by affecting the compound’s affinity for its biological targets. The higher efficacy of these long-chain compounds against Gram-negative bacteria could be explained by their structure: their slightly higher lipophilicity may facilitate entry through the outer membrane, which, along with a thinner peptidoglycan layer, represents a less polar barrier (Katsura et al., 2001; Vollmer and Seligman, 2010).

### 3.3 *In silico* pharmacokinetic properties

To complement these experimental findings, the pharmacokinetic properties were analyzed *in silico* using the SwissADME platform (Table 3). The LogP values (ranging from 4.92 for compound 3 to 9.42 for compound 13) confirmed the highly hydrophobic nature of all the derivatives. Although a high LogP can improve membrane permeability, it often compromises solubility. Notably, compounds 3, 4, 5, 6, and 9 showed a high predicted gastrointestinal (GI) absorption, which is a key parameter that determines the fraction of a drug that reaches systemic circulation (Vertzoni et al., 2019). In contrast, other derivatives showed low GI absorption, which is consistent with their lower predicted solubility.

**TABLE 3:** Pharmacokinetic parameters.

Compound	Log*P*_o/w_	Water solubility	GI	BBB permeant	P-gp substrate	Log*K*_p_
3	4.92	Moderately soluble	High	Yes	No	−3.52 cm/s
4	5.25	Moderately soluble	High	Yes	Yes	−3.70 cm/s
5	5.61	Moderately soluble	High	Yes	Yes	−3.45 cm/s
6	6.19	Moderately soluble	High	No	Yes	−2.96 cm/s
7	6.65	Poorly soluble	Low	No	Yes	−2.68 cm/s
8	6.25	Poorly soluble	Low	No	Yes	−2.98 cm/s
9	5.34	Moderately soluble	High	No	No	−3.37 cm/s
10	5.85	Moderately soluble	Low	No	Yes	−3.07 cm/s
11	6.23	Poorly soluble	Low	No	Yes	−2.71 cm/s
12	6.17	Moderately soluble	Low	No	Yes	−3.0 cm/s
13	9.42	Poorly soluble	Low	No	No	−0.83 cm/s
14	6.48	Poorly soluble	Low	No	Yes	−2.63 cm/s
15	8.12	Poorly soluble	Low	No	No	−1.46 cm/s
Ciprofloxacin	1.10	Soluble	High	No	Yes	−9.09
Meropenem	−0.37	Very soluble	Low	No	No	−10.31

Log*P*_o/w_, logarithm of the octanol/water partition coefficient (P); GI absorption, gastrointestinal absorption; BBB permeant, permeability of the blood–brain barrier; P-gp substrate, transport by P-glycoprotein; Log*K*_p_, logarithm of the cutaneous permeability coefficient.

Of the compounds analyzed, only compounds 3, 4, and 5 showed predicted permeability across the blood–brain barrier (BBB) (Table 3). Although high BBB permeability is often considered advantageous for drugs targeting neurological diseases, it is not directly relevant to the therapeutic scope of this study. In this case, the importance of this parameter lies primarily in potential systemic toxicity, as accumulation within the central nervous system (CNS) may pose safety concerns (Wu et al., 2023).

Compounds 3, 9, 13, and 15 tested negative for P-glycoprotein (P-gp) transport activity, which suggests they are not actively recognized or transported by this efflux protein. The lack of P-gp recognition could improve the absorption and tissue penetration of these compounds. However, it could also increase the risk of drug accumulation in specific tissues, which may lead to increased systemic toxicity (Veiga-Matos et al., 2023). The LogKp values were negative in all cases, ranging from −0.83 cm/s for compound 13 to −3.7 cm/s for compound 4. This indicates low cutaneous permeability and suggests limited potential for transdermal administration.

When compared with the reference antibiotics such as ciprofloxacin and meropenem, clear differences were observed. Ciprofloxacin and meropenem presented markedly lower LogP values (1.10 and −0.37, respectively), which is in line with their higher solubility in aqueous media. This contrasts with the moderate-to-low solubility of the synthetic derivatives, highlighting a potential formulation challenge. On the other hand, ciprofloxacin displayed extremely low skin permeability (LogKp −9.09 cm/s), whereas the synthetic derivatives exhibited higher permeability, which could be advantageous in contexts outside systemic oral administration. These comparisons emphasize that although classical antibiotics possess favorable solubility and lower lipophilicity, the derivatives studied here may require formulation strategies to optimize delivery.

Toxicological parameters predicted through the ADMETlab 2.0 server are shown in Table 4. None of the compounds exceeded 40% in hERG blockade, values generally associated with intermediate risk of cardiotoxicity. Although not alarmingly high, this suggests that experimental validation is essential to confirm the absence of cardiac safety issues (Su et al., 2021).

**TABLE 4:** Prediction of toxicological parameters.

Compound	hERG Blockers (%)	Ames (%)	DILI (%)	LD_50_ -log (mol/kg)	Lipinski ruler
3	37.35	36.08	38.99	2.16	Yes; 1 violation: LogP>4.15
4	38.74	38.41	37.75	1.95	Yes; 1 violation: LogP>4.15
5	38.43	35.63	38.95	1.95	Yes; 1 violation: LogP>4.15
6	40.18	38.89	41.91	2.15	Yes; 1 violation: LogP>4.15
7	40.71	35.92	43.25	2.12	Yes; 1 violation: LogP>4.15
8	40.47	36.19	44.61	2.04	Yes; 1 violation: LogP>4.15
9	38.75	39.29	35.92	2.03	Yes; 1 violation: LogP>4.15
10	37.62	37.78	49.96	1.91	Yes; 1 violation: LogP>4.15
11	38.28	35.14	44.39	2.2	Yes; 1 violation: LogP>4.15
12	39.18	40.1	42.08	2.08	Yes; 1 violation: LogP>4.15
13	40.8	37.23	56.84	2.3	Yes; 1 violation: LogP>4.15
14	39.1	35.44	46.08	2.27	Yes; 1 violation: LogP>4.15
15	40.61	37.29	55.2	2.24	Yes; 1 violation: LogP>4.15
Ciprofloxacin	43.52	46.2	49.08	2.33	Yes; 0 violations
Meropenem	37.53	43.39	58.89	2.17	Yes; 0 violations

hERG Blockers %, the human ether-a-go-go-related gene; Ames %, Ames test for mutagenicity; DILI %, drug-induced liver injury.

The Ames % values, which estimate mutagenicity, remained below 40% for all compounds, indicating a low probability of genotoxicity (Muñoz-Carrillo et al., 2025). Regarding hepatotoxicity, DILI % values ranged from 35.92% (compound 9) to 56.84% (compound 13). Although these values are below high-risk thresholds, they still indicate that potential hepatotoxicity should be monitored, particularly for compound 13. LD_50_ predictions indicated moderate acute toxicity, with compound 10 showing the lowest LD_50_ (1.91 mol/kg), thus potentially being the most acutely toxic derivative.

With respect to drug-likeness, all compounds complied with four of the five Lipinski rules, the only violation being their elevated LogP values (>4.15). This excess lipophilicity is likely to hinder the aqueous solubility and oral bioavailability. Nevertheless, such limitations can often be addressed through advanced formulation approaches, including lipid-based carriers, cyclodextrin inclusion complexes, or nanoparticle systems, which have been applied widely to improve the pharmacokinetic behavior of poorly soluble drugs (Felippe et al., 2025).

From a clinical perspective, these results suggest that the synthetic derivatives exhibit acceptable *in silico* safety profiles, with low mutagenic potential and intermediate hepatotoxicity and cardiotoxicity risks. However, the predictive nature of ADMET models must be emphasized; these are computational estimations, not experimental measurements, and are, therefore, subject to uncertainty. Algorithms such as SwissADME and ADMETlab 2.0 rely on statistical models derived from large datasets, which provide valuable first insights but cannot fully capture the complexity of *in vivo* metabolism, distribution, or toxicity (Veiga-Matos et al., 2023). Consequently, these findings should be interpreted with caution and validated through preclinical testing.

Overall, the *in silico* pharmacokinetic and toxicological analysis of the derivatives highlights both opportunities and challenges. High GI absorption in certain compounds (3, 4, 5, 6, and 9) is promising, whereas the observed Lipinski violations point to the need for formulation strategies to enhance the solubility and bioavailability. Predicted toxicity values indicate manageable risks, although experimental validation will be critical before advancing to biological assays. In summary, although these derivatives show therapeutic potential, their further development will depend on optimizing pharmacokinetic profiles, minimizing toxicity, and addressing formulation challenges to ensure clinical applicability. Finally, the results confirm that the selected synthetic pathway is viable and yields compounds with promising biological activity. Furthermore, these findings open the door to future studies focused on optimizing reaction conditions, improving yields, and expanding the structural diversity of the derivatives. Additional biological evaluations, including *in vitro* and *in vivo* assays, are necessary to further validate the antibacterial potential of these compounds. It is also important to consider the scalability of the synthesis and the environmental impact of the process, especially if these compounds are to be developed as pharmaceutical candidates. Overall, in this work, we highlight the value of natural products as platforms for the design of novel bioactive molecules through hemisynthetic approaches.

## 4 Conclusion

In conclusion, ester-type bakuchiol derivatives demonstrated superior antibacterial activity compared to their ether-type analogs. Within this group, compounds 4 and 5 were the most effective against Gram-positive bacteria, whereas compounds 6 and 7 exhibited superior potency against *P. aeruginosa*, a clinically relevant Gram-negative pathogen. The *in silico* analysis supports the activity of these compounds as antibacterial agents, showing favorable gastrointestinal absorption and moderate toxicity profiles. However, their high LogP values and low solubility represent a challenge that will require formulation strategies to optimize their pharmacokinetic properties. Collectively, these results identify compounds 4, 5, 6, and 7 as leading candidates for future *in vivo* studies and synthetic optimizations.

## Data Availability

The original contributions presented in the study are included in the article/Supplementary Material; further inquiries can be directed to the corresponding authors.
