# Supplementary material for: Synthetic bakuchiol derivatives: ester and ether analogs with activity against clinically important bacteria
Source: Front Pharmacol. 2025 Oct 27;16:1619997. doi: 10.3389/fphar.2025.1619997 (PMC12597907; doi:10.3389/fphar.2025.1619997)

Supplementary materials

^{1}H spectra of compound **6**.

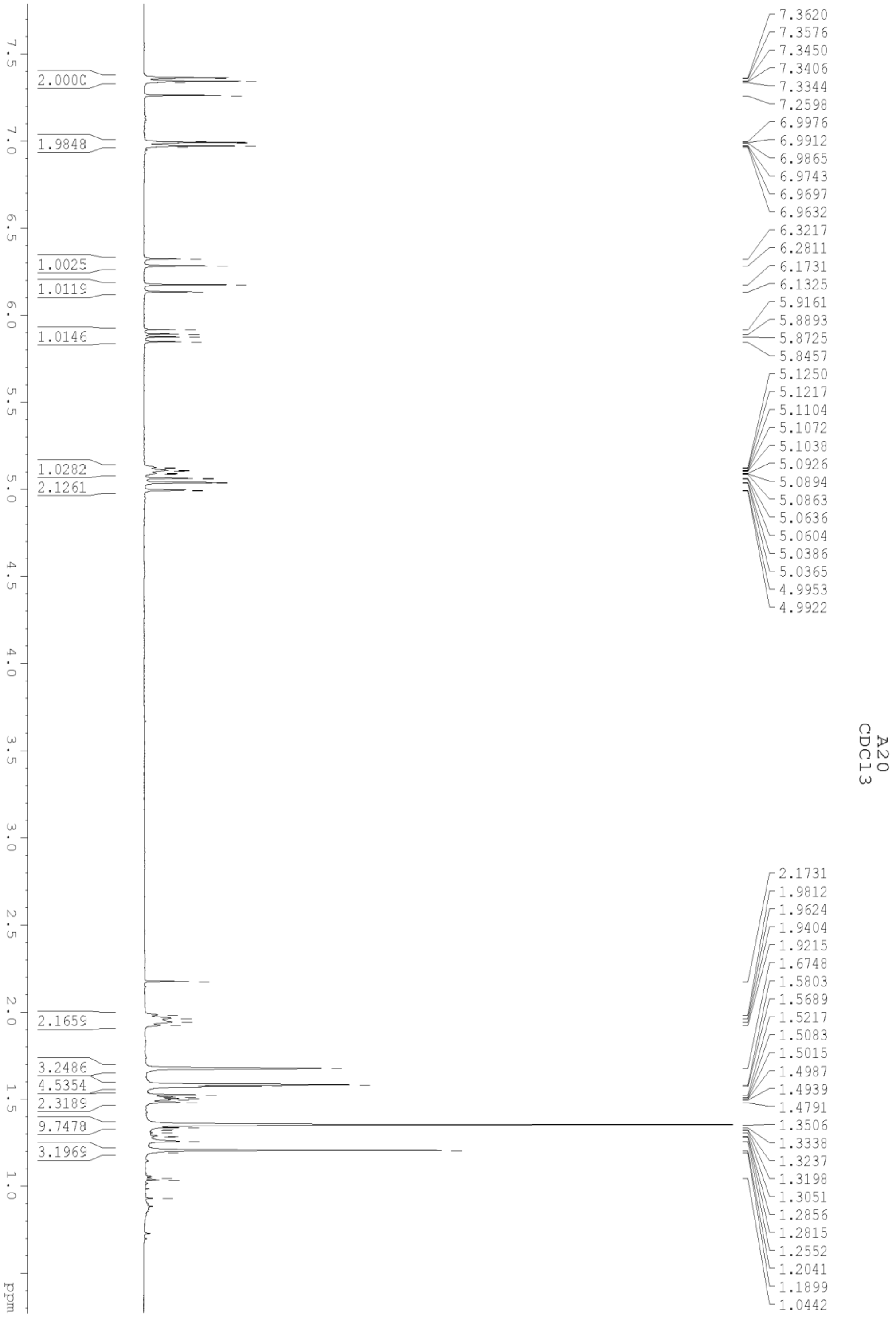

^{13}C spectra of compound **6**.

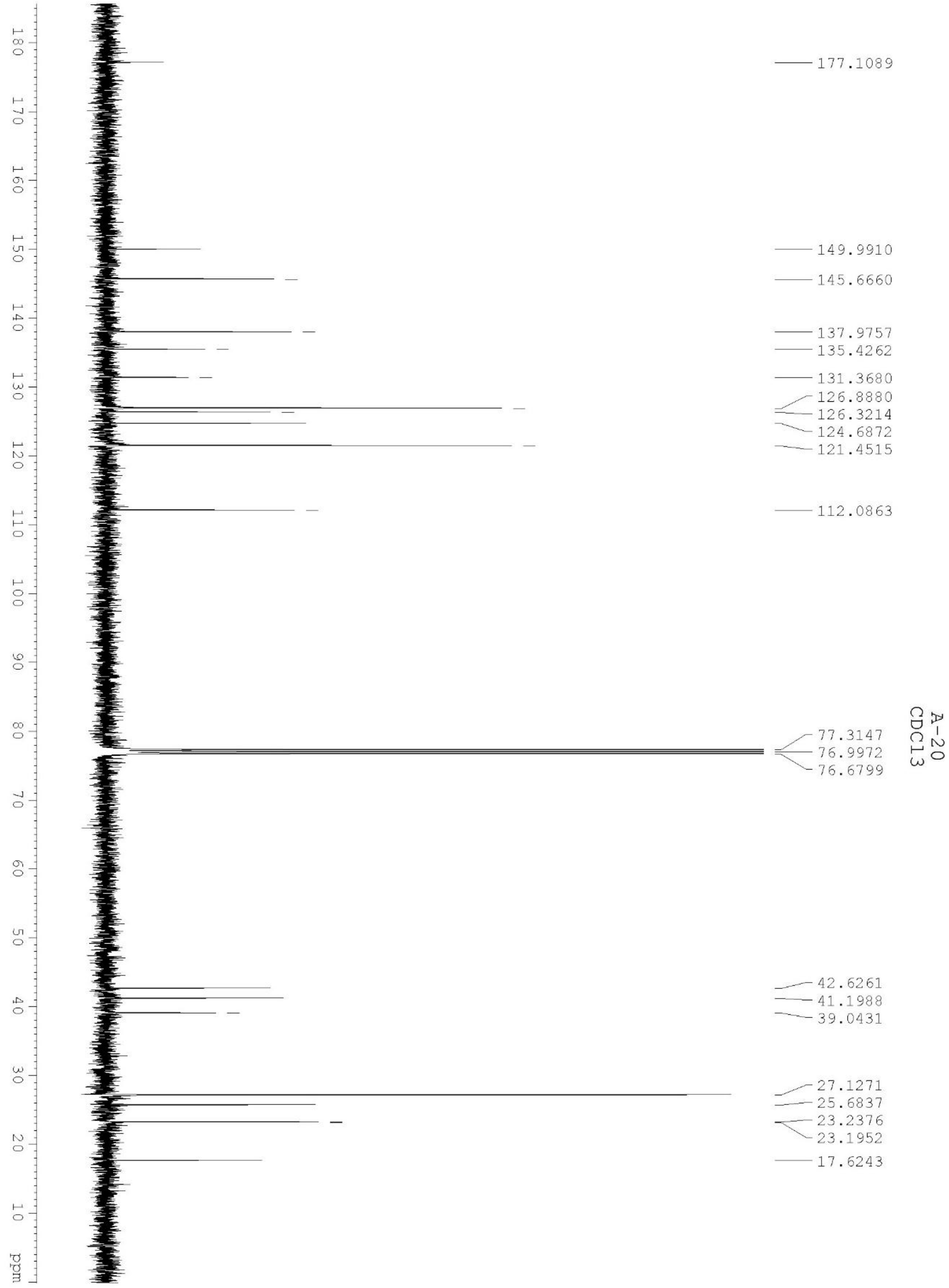

^{1}H spectra of compound **10**.

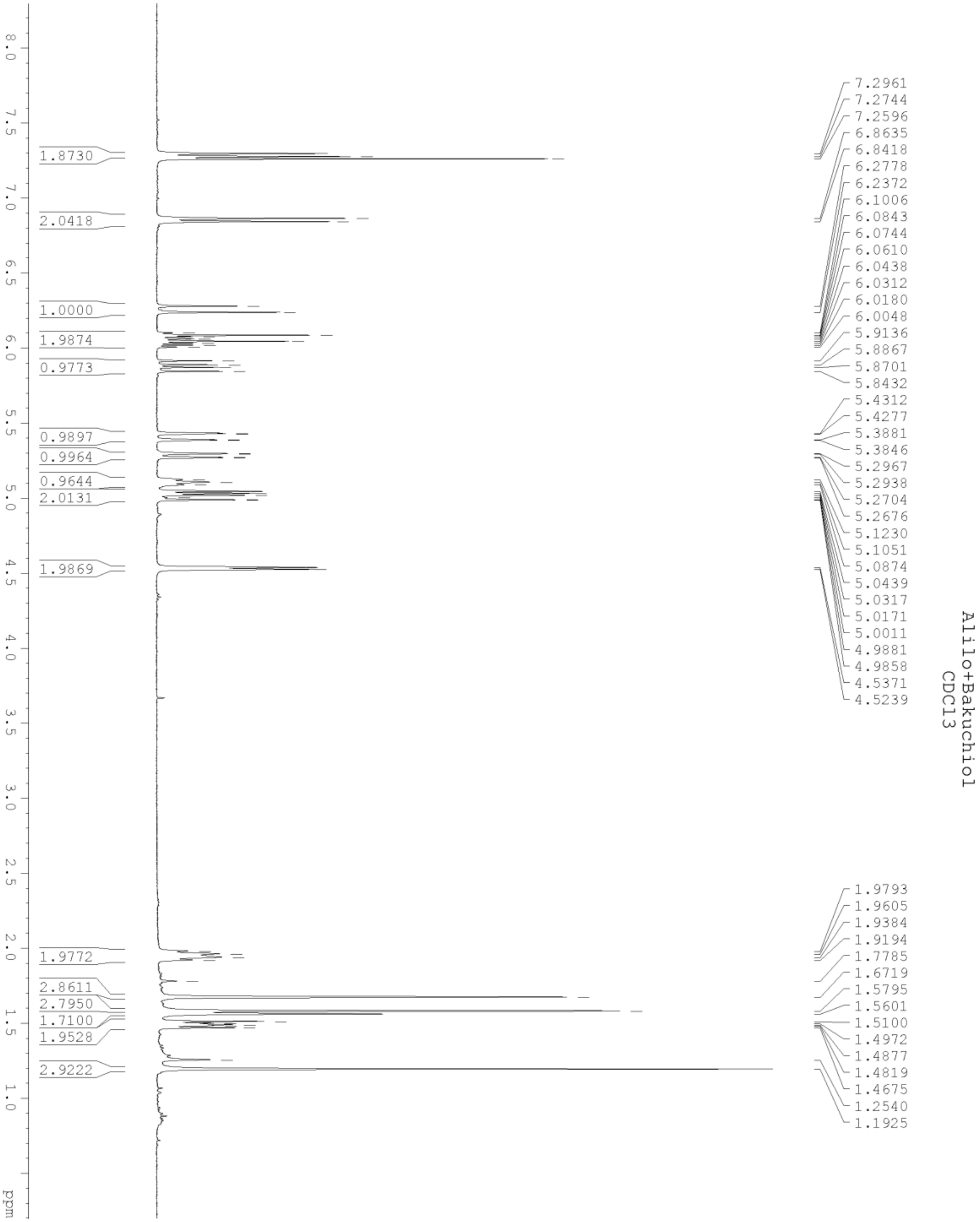

^{13}C spectra of compound **10**.

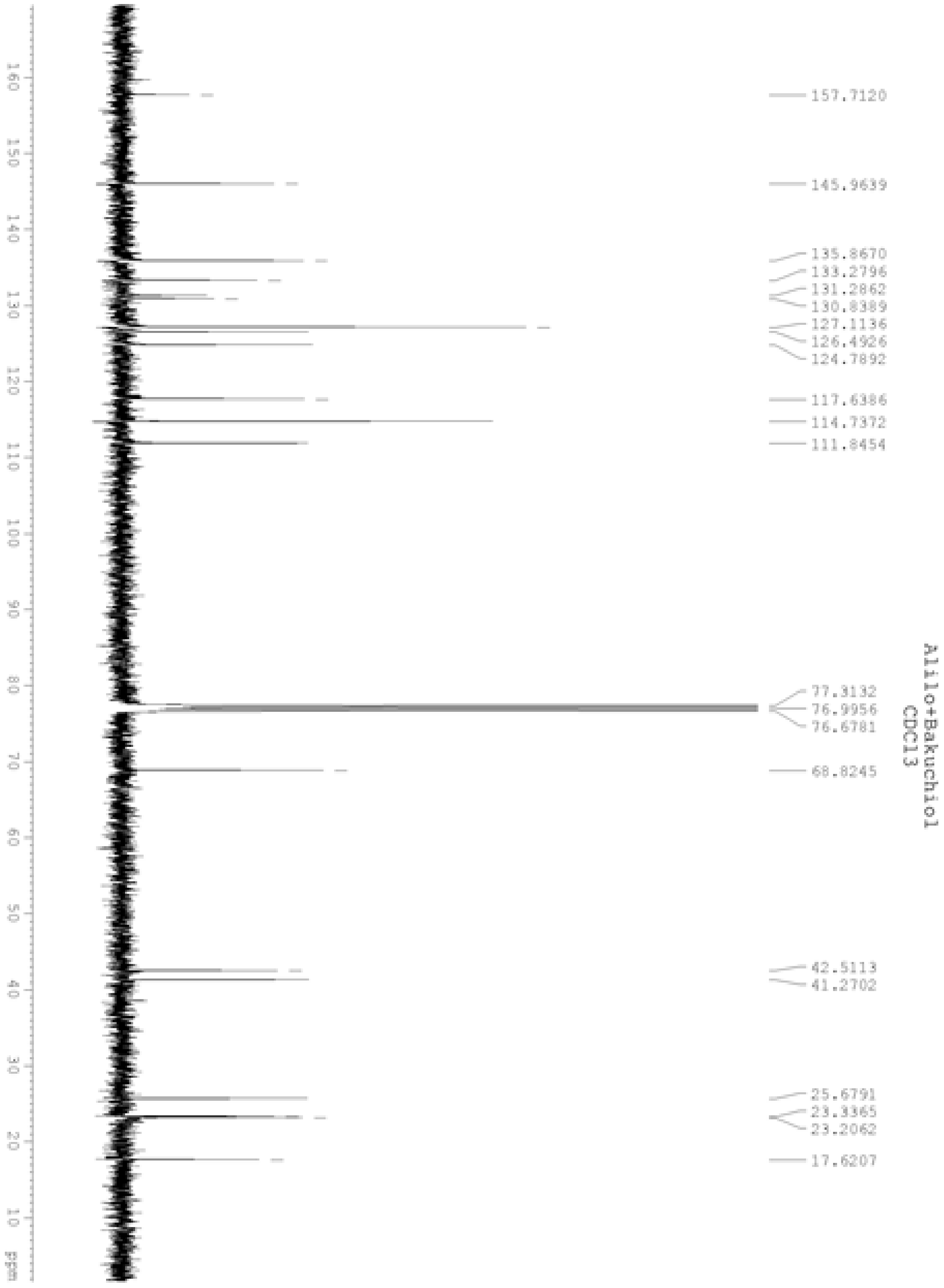

^{1}H spectra of compound **11.**

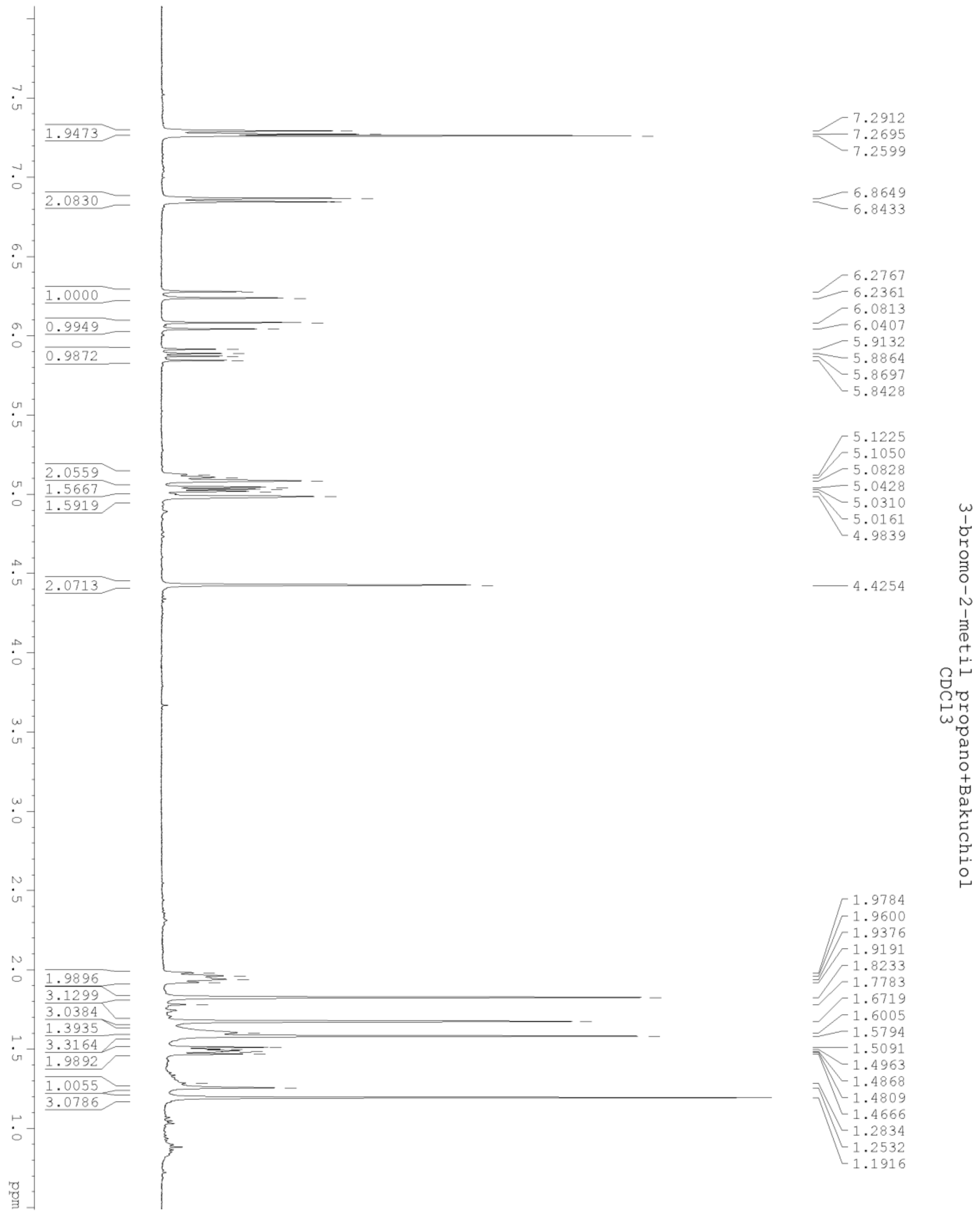

^{13}C spectra of compound **11.**

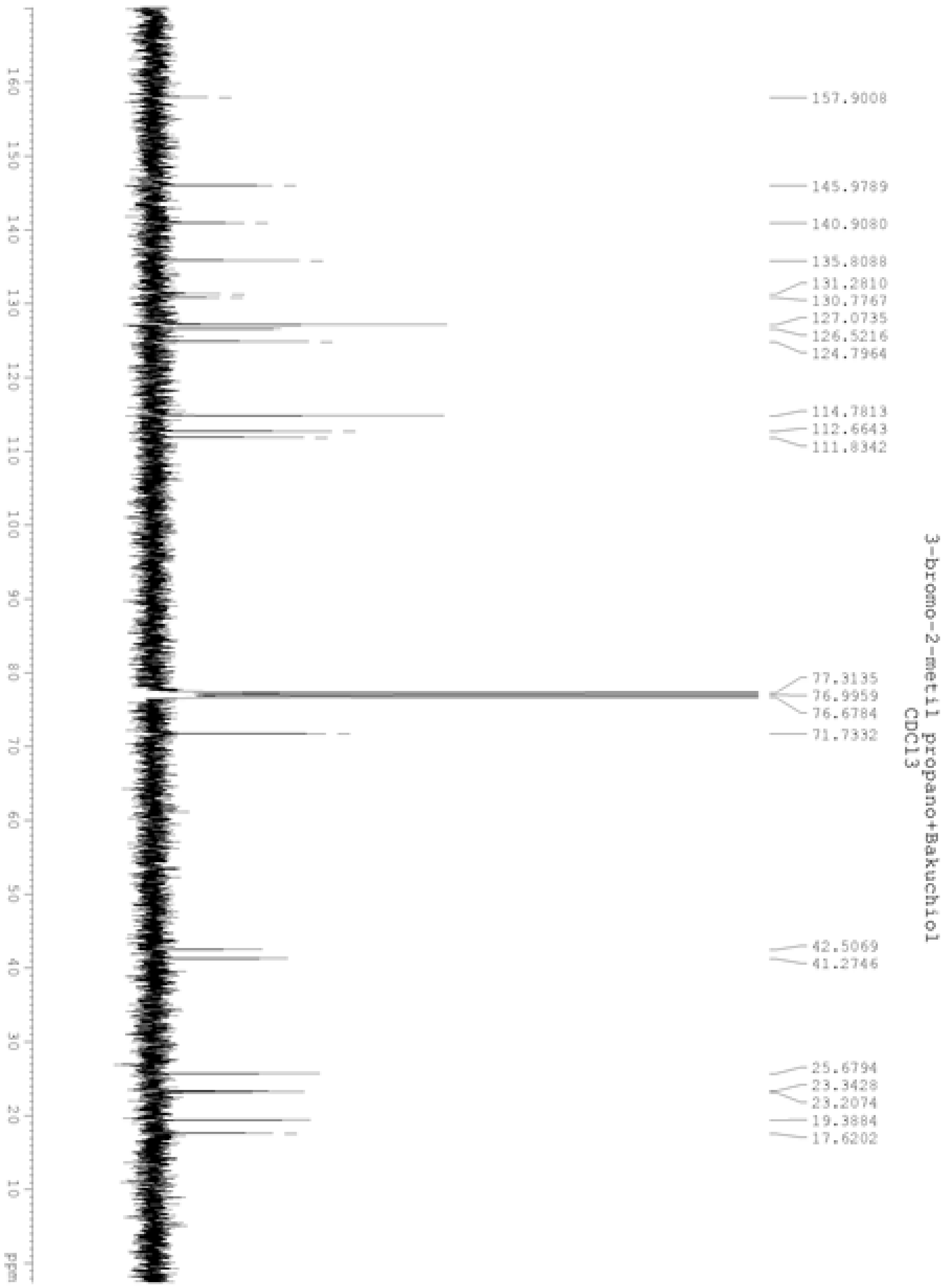

^{1}H spectra of compound **12.**

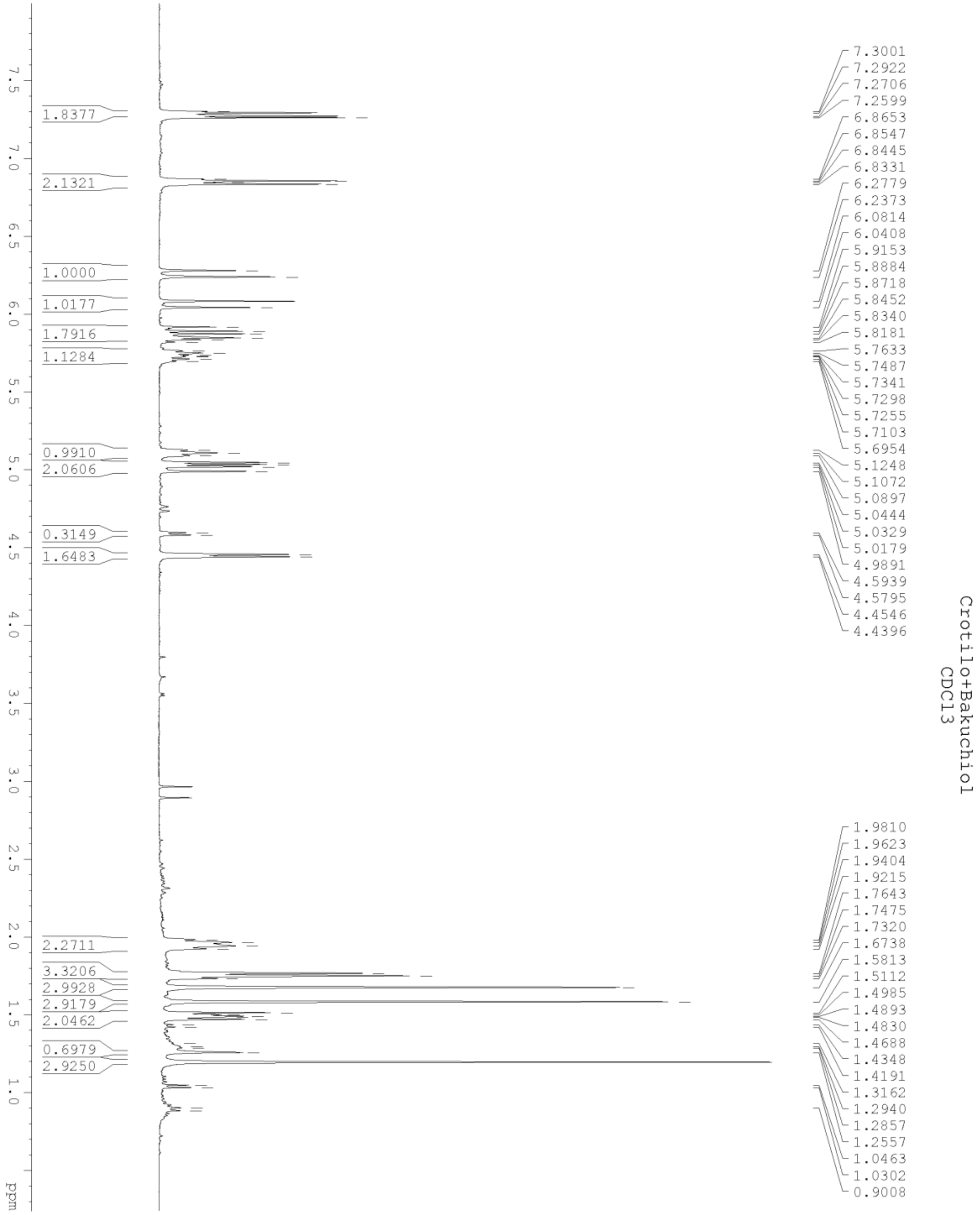

^{13}C spectra of compound **12**.

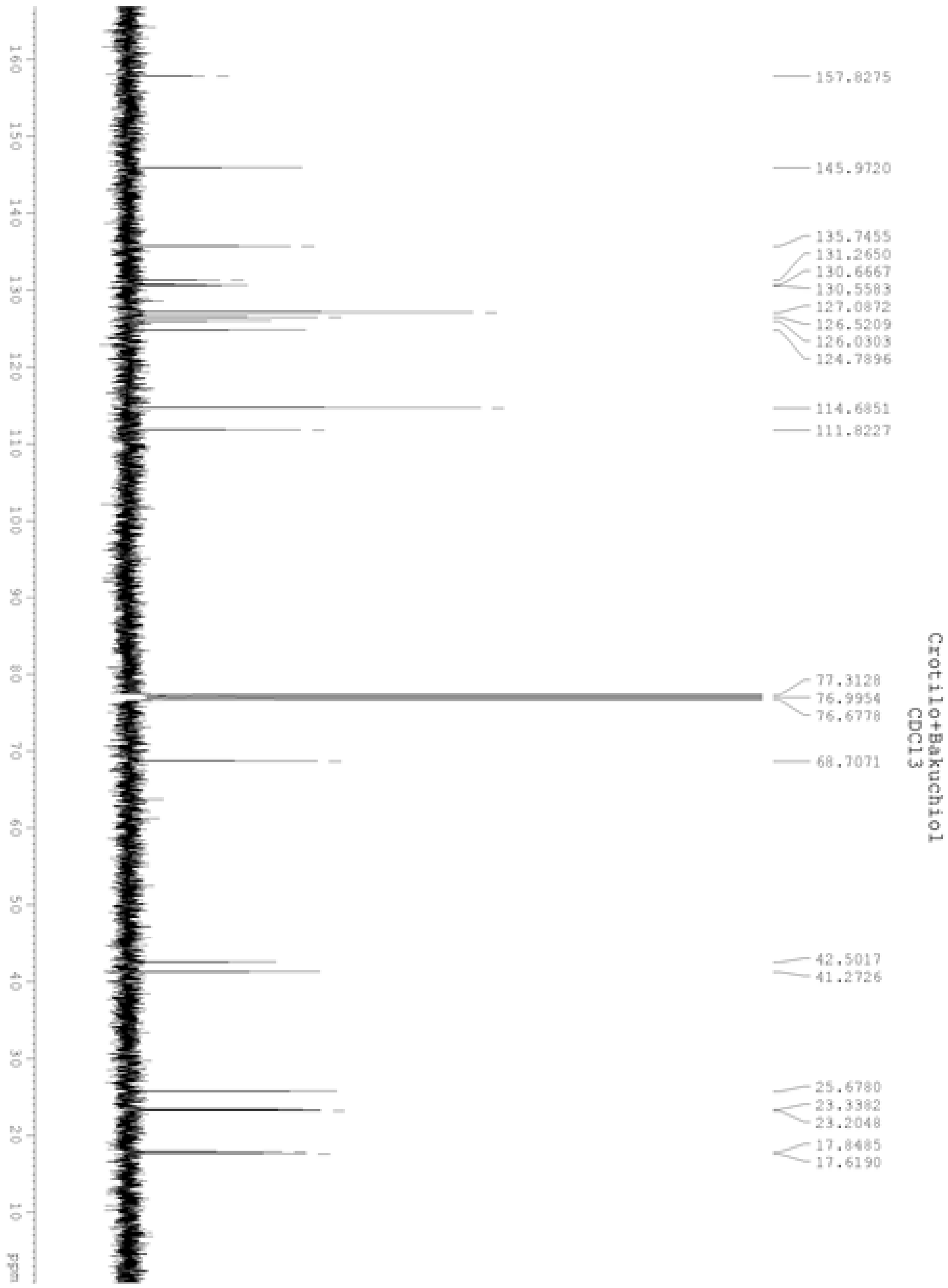

^{1}H spectra of compound **13**.

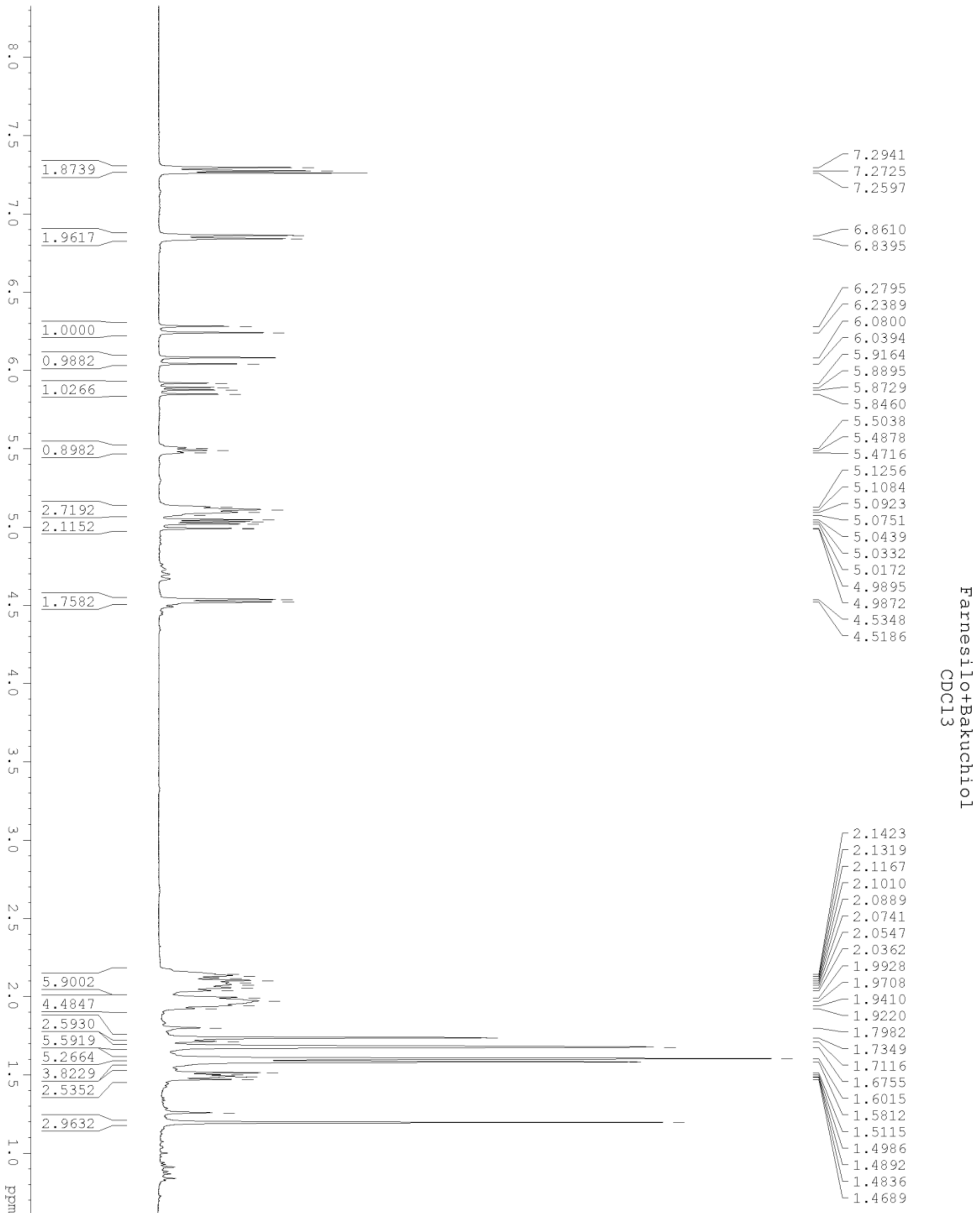

^{13}C spectra of compound **13.**

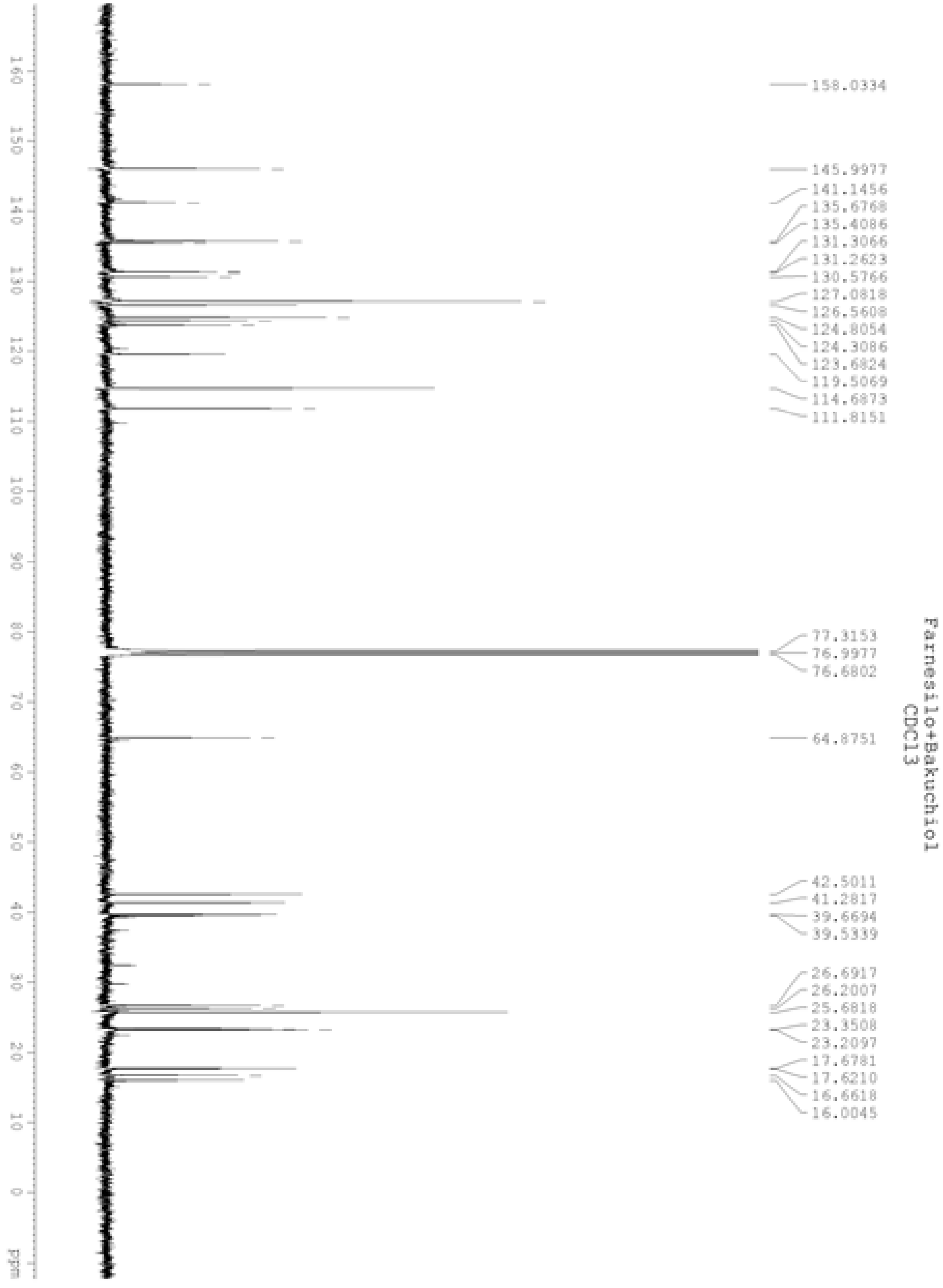

^{1}H spectra of compound **14.**

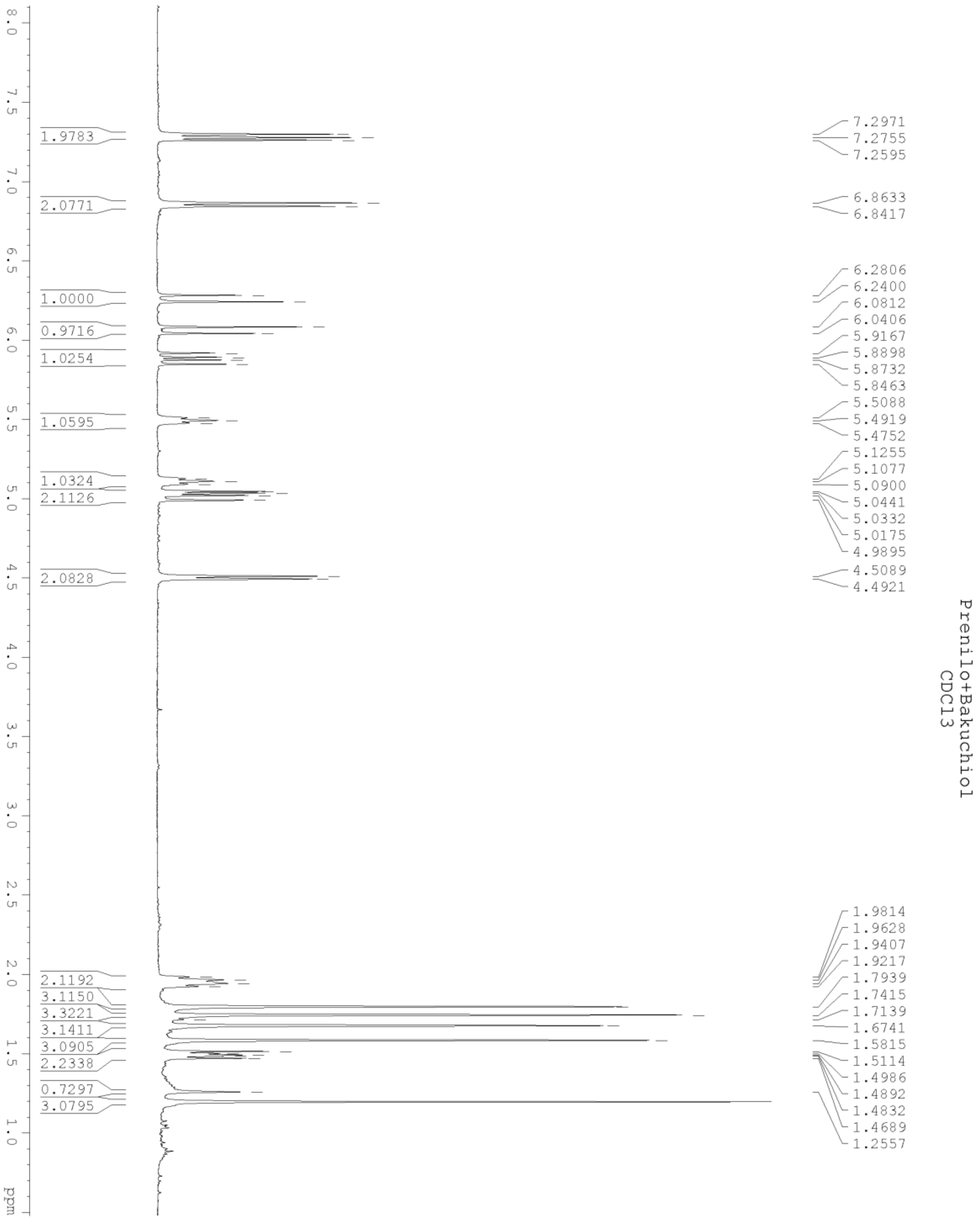

^{13}C spectra of compound **14.**

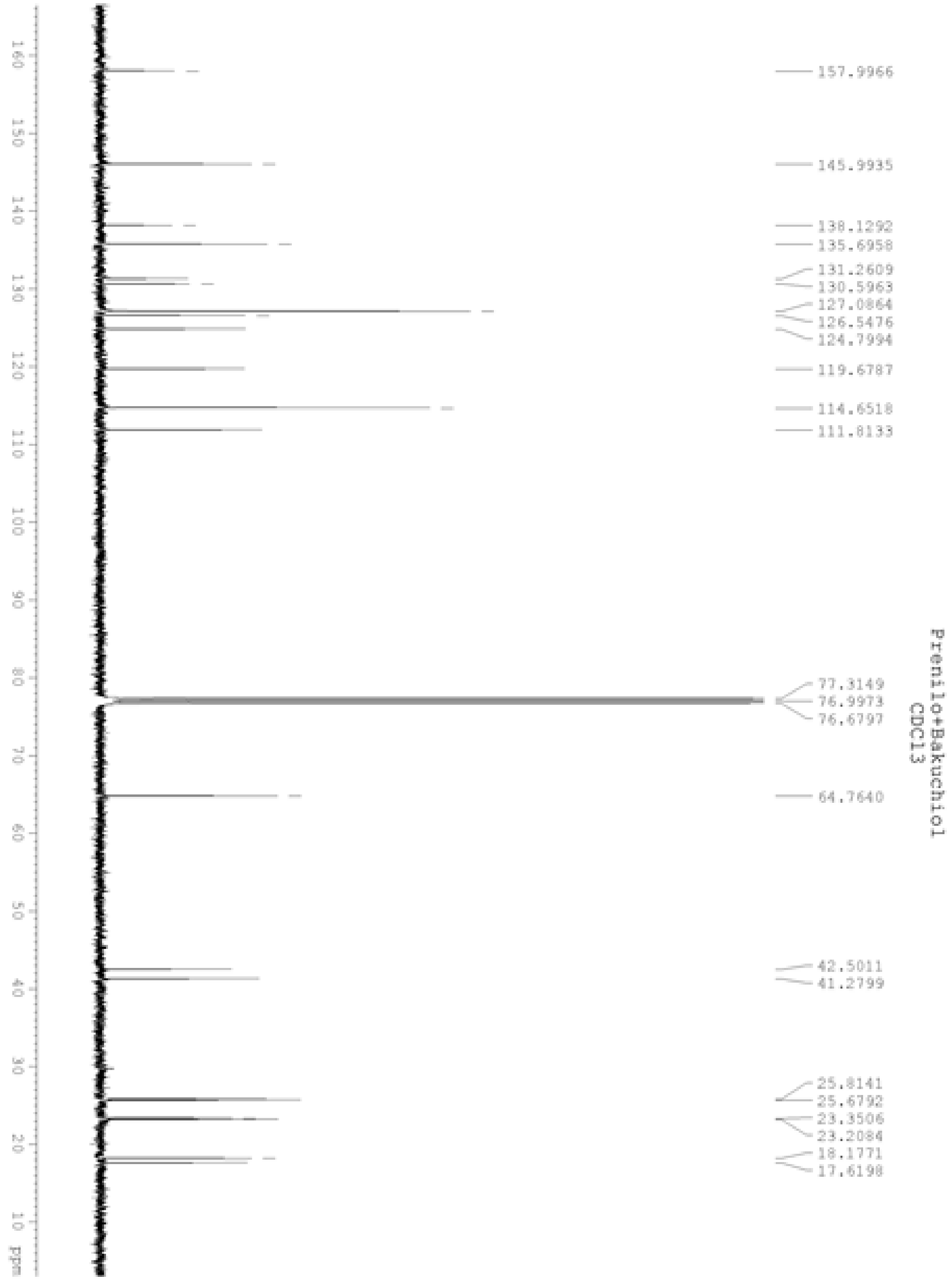

^{1}H spectra of compound **15.**

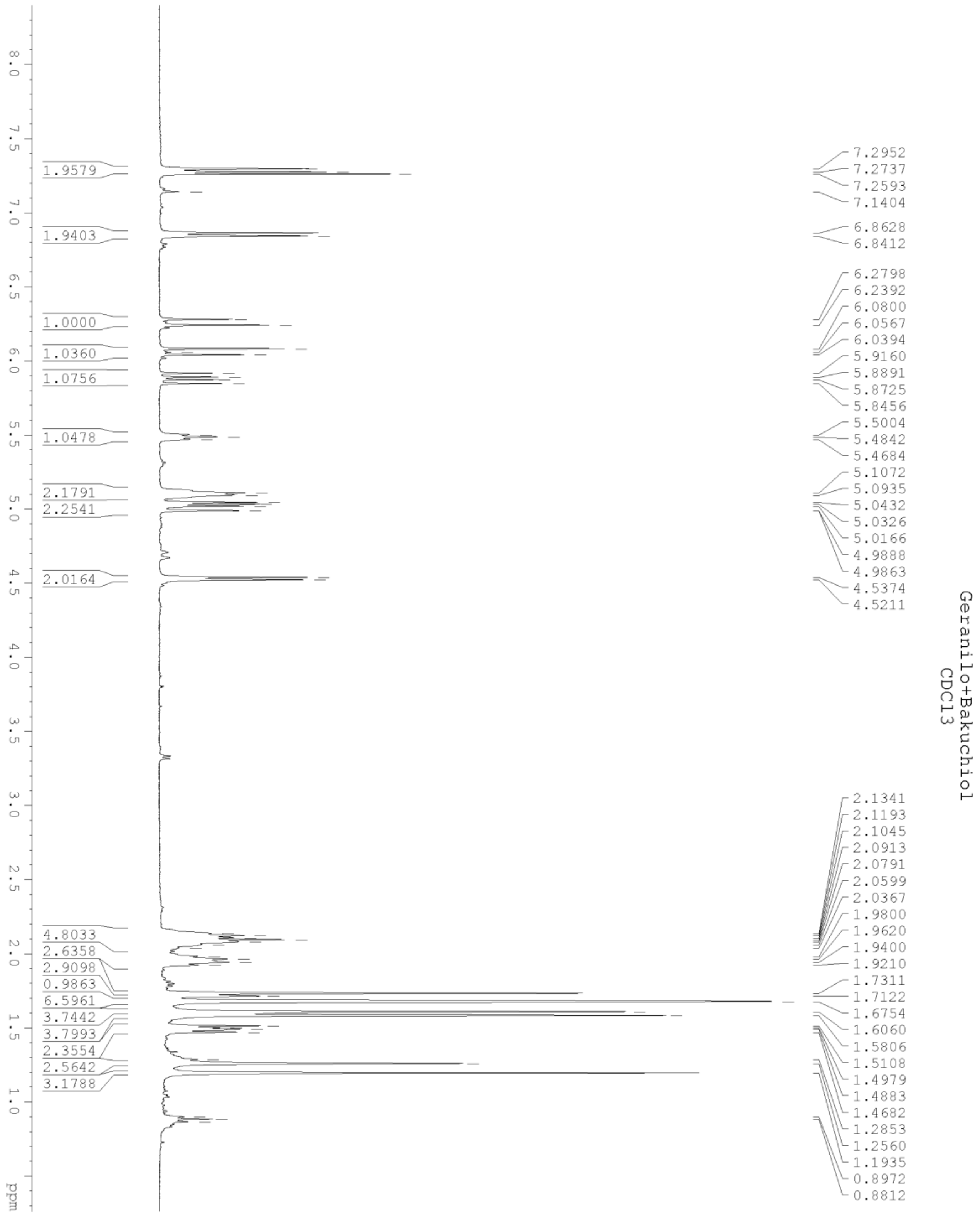

^{13}C spectra of compound **15**.

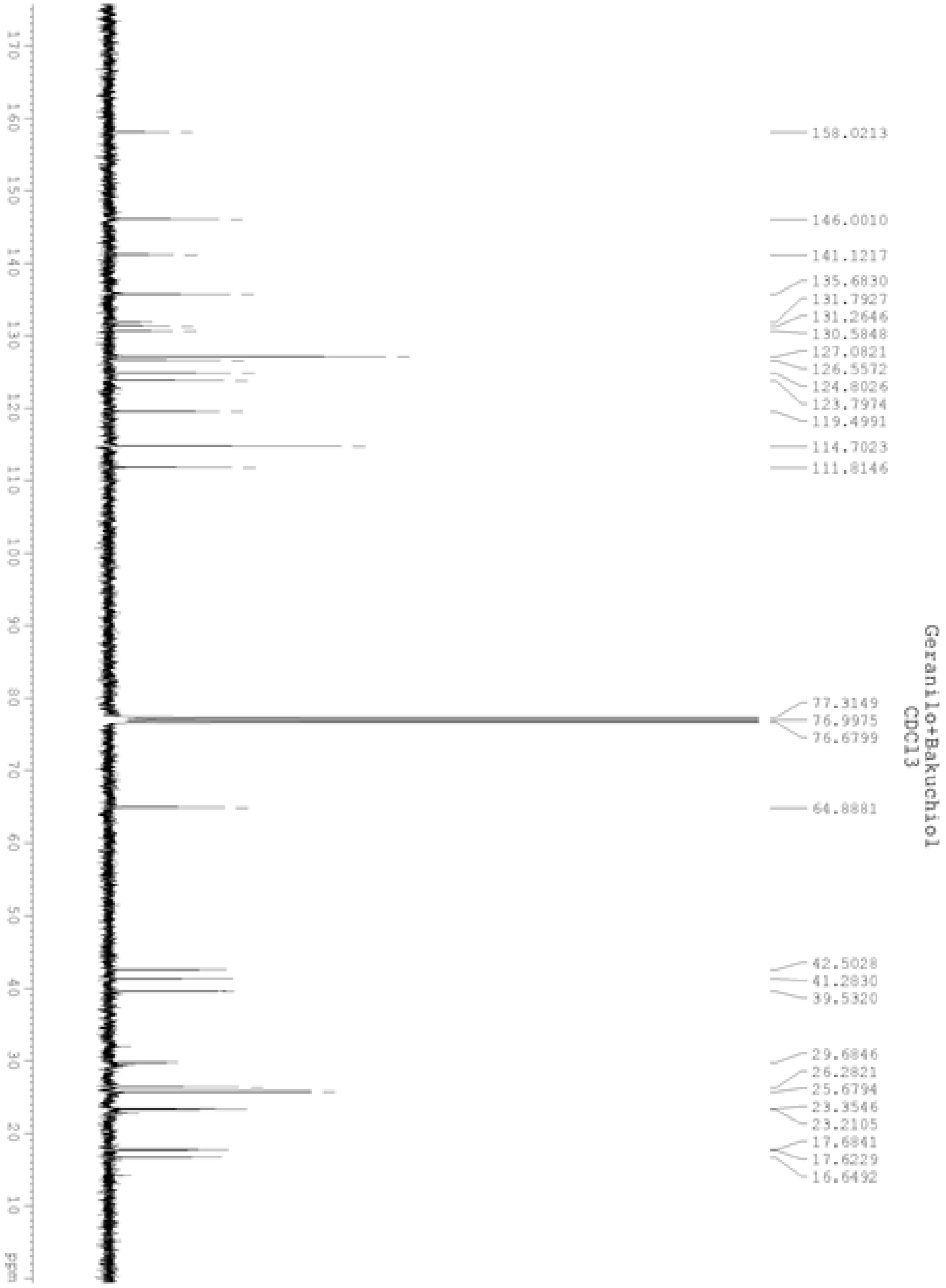

Supplement: Supplementary file 1 [file DataSheet1.pdf]
